# The hypoglycemic effect of *Juglans regia* leaves aqueous extract in diabetic patients: A first human trial

**DOI:** 10.1186/2008-2231-22-19

**Published:** 2014-01-21

**Authors:** Saeed Hosseini, Hasan Fallah Huseini, Bagher Larijani, Kazem Mohammad, Alireza Najmizadeh, Keramt Nourijelyani, Leila Jamshidi

**Affiliations:** 1 Endocrinology and Metabolism Research Center, Endocrinology and Metabolism Clinical Sciences Institute, Tehran University of Medical Sciences, Tehran, Iran; 2 Pharmacology and Applied Medicine Department of Medicinal Plants Research Center, Institute of Medicinal Plants, ACECR, Karaj, Iran; 3 Department of Epidemiology and Biostatistics, Tehran University of Medical Sciences, School of Public Health, Tehran, Iran; 4 Karaj Diabetes Society, Alborz, Karaj, Iran; 5 Department of Nutrition, Tehran University of Medical Sciences, School of Public Health, Tehran, Iran

**Keywords:** *J. regia*, Insulin, Diabetes mellitus, Glucose, Liver enzymes

## Abstract

**Background:**

*Juglans regia* L. (*J. regia* ) is one of the medicinal plants traditionally used for treatment of diabetes in Iranian medicine. The effect of this plant has already been investigated on animal models; however, this is the first study conducted on human subjects. The aim of this study is to investigate the hypoglycemic effect of *J. regia* leaves aqueous extract in type 2 diabetes patients. Fifty eight Iranian male and female patients with type 2 diabetes were enrolled. The patients were randomly allocated into two groups. One group (n = 30) received *J. regia* leaves extract while the other group (n = 28) received placebo. Fasting blood samples were collected at the beginning of the study and after two months for determination of HbA1c and blood glucose level as a main outcome and insulin, SGOT, SGPT, and ALP level as secondary outcome.

**Results:**

Our analysis showed that serum fasting HbA1C and blood glucose levels were significantly decreased and the insulin level was increased in patients in the *J. regia* arm.

**Conclusions:**

The results indicate that *J. regia* aqueous extract favorably affects blood levels of glucose, insulin and HbA1C in type 2 diabetic patients.

## Introduction

Diabetes mellitus is a major public health problem in both developed and developing countries. Among the leading causes of death, diabetes mellitus is ranked seventh and when fatal complications are taken into the account, it is ranked third [1]. Recently, there has been increasing interest in the use of medicinal plants. Frequently, however, it is mandatory to provide scientific proof in order to justify the use of a plant or its active components [2]. Several studies confirm the potential therapeutic efficacy of some medicinal plants in treatment of diabetic patients [3]. Since natural products may provide better treatments with fewer side effects than the existing artificial medications, a major focus for suitable anti-hyperglycemic agents has been on plants used in traditional medicine [4]. The probable efficiency of medicinal plants for treating diabetes and their abundance in most parts of the world facilitate their usage. *J. regia* belongs to the family Juglandaceae. It includes 3 species: *J. nigra*, *J. cinerea*, and *J. regia* although only *J. regia* type is found in Iran [5]. Investigations show that *J. regia* extract contains ellagitannins which contains anti-cancer agent and with anti-inflammatory properties [6]. The key chemical composition of walnut is Juglone (5-hydroxy-1,4-naphthoquinone), the toxic compound which is found only in green and fresh walnuts, but such property disappear in dried leaves [7]. Other several phenolic compounds with antioxidant properties have been identified in *J. regia* leaves [8]. In an experimental study treatment of *J. regia* extracts in experimental animals resulted in a significant decrease in blood glucose, glycosylated hemoglobin, LDL, triglyceride and total cholesterol and a significant increase in insulin and HDL level [9]. In another study the favorable effects of *J. regia* leaves on pancreatic cells in alloxan induced diabetic rat have been reported [10]. In addition, Asgary and co-authors showed that fasting blood sugar decreased meaningfully where as insulin level increased and glycosylated hemoglobin decreased significantly in diabetic groups receiving either glibenclamide or *J. regia* extract compared with the diabetic untreated group [11]. In the present study, we evaluated the effect of *J. regia* leaves aqueous extract on type II diabetic patients for the first time.

## Methods

### Plant material and extraction procedure

*J. regia* leaves were collected from institute of medicinal plant Farm Karaj Iran. The leaves were washed by distilled water and placed in oven at 37°C to dry. Then, the leaves were completely pulverized using a porcelain mortar and pester. The powder was soaked in distilled water and placed on a magnetic stirrer for 24 hours to resolve completely. The resulting solution was passed through a filter paper and dried under appropriate conditions (in the oven at 37°C).

### Participants

**Figure 1:**
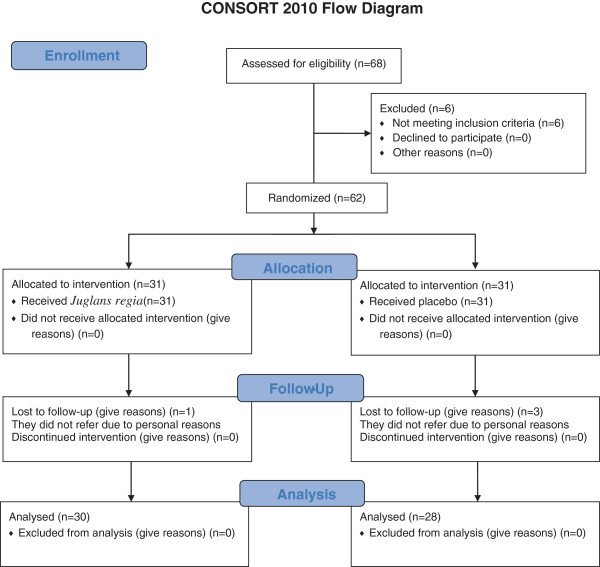
The CONSORT flowchart describing the progress of the patients through the trial.

Initially, total of 62 patients (37 female and 25 male) with type 2 diabetes were recruited through an announcement made in the Diabetes Clinic of Shariati Hospital. The patients were randomly divided into two equal groups (*J. regia* and placebo). Shortly after the start of the study, 4 patients withdrew for personal reasons and the study continued with the remaining 58 patients (Figure 1) including 35 female and 23 male. The research protocol was approved by the Ethics Committee of the Endocrinology and Metabolism Research Center of Shariati Hospital and written informed consent was obtained of each patient prior to the study. All the patients who participated were aged between 40 and 65 years and had a confirmed diabetes type 2 diagnoses according to ADA criteria [12]. The inclusion criteria were: age 30 to 80, HbA1C > 7% fasting blood glucose less than 250 mg/L and taking maximum dose of two anti- diabetic drug (metformin and glibenclamide). The exclusion criteria were: Immune deficiency, pregnancy and lactation, cardiovascular disease, currently receiving corticosteroids and thiazide, uncontrolled thyroid dysfunction, acute infection, history of diabetic ketoacidosis, Cr > 1.5 for male, Cr > 1.2 for female, acute hepatitis, cirrhosis, proliferative retinopathy and severe weight loss (at least 10% during last 6 months).

### Protocol

The patients were randomly allocated to two groups using a balanced randomization method. The patients and investigators who carried out clinical and para-clinical assessments were unaware of the treatment groups and the type of medication. *J. regia* (100 mg) and placebo tablets of the same shape were provided by the Institute of Medicinal Plants (Tehran, Iran). The conventional oral hypoglycemic agents (metformin and glibenclamide) continued in both groups. Compliance was assessed indirectly using the pill count method. The first group (32 patients) received one 200 mg *J. regia* tablet two times a day before meal. Also, the control group (29 patients) received a placebo tablet twice a day before meal. The blood levels of HbA1c and fasting blood glucose, insulin, SGOT, SGPT, and ALP were measured at the beginning and after 2 months of the study in both groups. Blood samples were drawn after overnight fasting (12 h). Fasting glucose levels were measured using the gloucose-oxidase method using a Beckman Glucose2 Analyzer immediately after blood sampling at the Endocrine Research Center Laboratory. Glycosylated hemoglobin levels were measured by a D-10 hemoglobin testing system (Bio-Rad Laboratories, Inc.). All other blood sample parameters were measured by an auto analyzer (Hitachi 902) using commercial kits. Patients were visited and examined every month and the efficacy of treatment was checked by measurement of the fasting blood glucose level. Sample size was calculated comparing the main outcome of the study (namely FBS) to detect 10 mg/dl of reduction from the baseline group with 80% of power and 5% sig. level.

### Statistical analysis

Paired sample t test was conducted for comparing the pre and post evaluation and independent sample t test was preformed to compare the differences between two groups. Statistical analysis was carried on using R statistical package version3.0.1 (R Core Team 2013. R: A language and environment for statistical computing. R Foundation for Statistical Computing, Vienna, Austria). A p-value less than 0.05 was considered statistically significant (Figure 1).

## Results

All 58 patients completed the study with no further dropouts. The patients' demographic characteristics are summarized in Table 1.

**Table 1:** **The demographic characteristics of patients in placebo and *J*. *regia *** **treated groups (mean ± SD)**

**Groups**	***J*. *regia***	**Placebo**	**p-value**
Age (year)	58.1 ± 4.2	56.2 ± 7.3	0.21
Sex (male/female)	11 M/19 F	12 M/16 F	0.69
Duration of disease (year)	5.2 ± 1.6	6.2 ± 1.1	0.006
Weight (kg)	62.3 ± 5.6	60.6 ± 6.1	0.52

The baseline findings of patients in the two groups (*J. regia* and placebo) were compared for FBS, HbA1c, insulin and liver enzymes. The two groups were completely comparable regarding baseline and demographic characteristics (Table 2).

**Table 2:** Para clinical characteristics of the two groups at the beginning of the study (mean ± SD)

**Groups**			
	***J*. *regia***	**Placebo**	**p-value**
**FBS (mg/dl)**	165 ± 52	168 ± 65	0.82
**HbA1c (%)**	8.5 ± 1.6	8.3 ± 1.7	0.588
**Insulin (ng/ml)**	0.9 ± 0.7	0.7 ± 0.4	0.274
**SGOT (U/L)**	11.7 ± 5.3	12.5 ± 4.2	0.910
**SGPT (U/L)**	8.7 ± 3.7	9.5 ± 3.1	0.665
**ALP (IU/L)**	159 ± 34	151 ± 59	0.502

The average fasting blood glucose level in the *J. regia* group was 165 ± 54 mg /dL in the beginning of the study which significantly decreased (p < 0.05) to 144 ± 65 mg /dL after 2 months of treatment with *J. regia*. The average fasting blood glucose level in the placebo group was not significantly changed after 2 months of the study.

The average HbA1c level in the *J. regia* group at the beginning of the study was 8.5 ± 1.6 mg /dL, which decreased significantly (p < 0.05) to 7.6 ± 1.3 mg /dL after 2 months of *J. regia* treatment. The average HbA1c level in the placebo group was 8.3 ± 1.7 mg /dL at the beginning of the study which decreased significantly (p < 0.05) to 7.7 ± 1.5 mg /dL after 2 months of placebo treatment.

The average insulin level in the *J. regia* group was 8.6 ± 4.2 mg /dL in the beginning of the study which increased significantly (p < 0.05) to 10 ± 4.7 mg /dL after 2 months of treatment with *J. regia*. The average insulin level in the placebo group was not significantly changed after 2 months of placebo treatment.

The results of the clinical findings in both groups in the beginning and after 2 months of the study are summarized in Table 3. Finally, No liver functional or other gastrointestinal side effects of the treatment were reported during the study.

**Table 3:** **The serologic parameters in Placebo and *J*. *regia *** **treated groups (mean ± SD)**

	***J*. *regia *** **(n = 30)**			**Placebo (n = 28)**			
	**Beginning**	**After 2 month**	***p*-value**	**Beginning**	**After 2 month**	***p*-value**	***P*-value Difference of difference**
FBS (mg/d L)	165 ± 54	144 ± 65	0.017	169 ± 71	171 ± 61	0.847	0.079
HbA1c (%)	8.5 ± 1.6	7.6 ± 1.3	0.000	8.3 ± 1.7	7.7 ± 1.5	0.042	0.068
Insulin (U/L)	8.6 ± 4.2	10.4 ± 4.7	0.007	8.6 ± 3.6	9.1 ± 3.9	0.413	0.138
SGOT (U/L)	11.6 ± 5.3	12.6 ±5.0	0.293	12.5 ± 4.2	12.2 ± 4.5	0.110	0.879
SGPT (U/L)	8.7 ± 3.7	9.7 ± 2.9	0.849	9.5 ± 3.1	8.5 ± 2.8	0.708	0.692
ALP (IU/L)	159 ± 30	163 ±43	0.469	152 ± 59	152 ± 46	0.228	0.660

HbA1c (%).

8.5% NGSP = 70 mmol/mol IFCC = 198 mg/dL eAG.

7.6% NGSP = 60 mmol/mol IFCC = 171 mg/dL eAG.

8.3% NGSP = 67 mmol/mol IFCC = 191 mg/dL eAG.

7.9% NGSP = 62 mmol/mol IFCC = 179 mg/dL eAG.

## Discussion

The results suggest that *J. regia* improves glycemic in the type 2 diabetic patients without any adverse effects on the kidney and hepatic function. The results showed that although there were no significant differences in the main parameters between the two groups of patients at the beginning of the study, *J. regia* extract treatment significantly lowered fasting blood glucose and HbA1c, and increased the insulin level in diabetic patients at the end of the study. The mechanism underlying the glucose lowering effect of *J. regia* might be due to increase release of insulin from remnant β-cells and/or regenerated β-cells, restore insulin sensitivity [10], interference with the absorption of dietary carbohydrates in the small intestine [13], and facilitate utilization of glucose by peripheral tissues mediated by an insulin dependent glucose transporter [14]. The key compound responsible for antihyperglycemic effect of the plant extract may be phenolic substances, such as gallic acid and caffeoylquinic acid [15]. Phenolic acids and flavonoids are two groups major phenolic compounds existing *J. regia* leaves [8]. Caffeoylquinic acid and acid coumaroylquinic are major phenolic acids in *J. regia* leaves. The main flavonoids in walnut leaves are juglone, quercetin 3-galactoside, quercetin 3-arabinoside, quercetin 3-xyloside, quercetin 3-rhamnoside and quercetin 3-pentoside [16,17]. Histomorphologic studies conducted in animal models of liver sections showed that the diabetic group had moderate inflammation in the portal space, and mild lobular necrosis was also observed where as in diabetic rats treated with the walnut extract, very mild portal inflammation and inflammatory cells were seen without any significant intra-lobular necrosis [18,19]. The same study showed that in diabetic rats treated with walnut leaf extract, serum AST and ALT had a significant decrease when compared to diabetic rats. The decrease in the plasma levels of AST and ALT is due to the decrease in liver cells injury, glucose, cholesterol, triglyceride, hepatic lipid levels and subsequently prevention from the formation of fatty liver. Our findings also indicated that treatment with *J. regia* did not adversely affects SGOT and SGPT levels as indication of its safety. In conclusion, our finding showed that the use of *J. regia* leaves was effective for glycemic control of diabetic patients. Since this study was conducted on human samples for the first time, therefore the lowest dose of the drug was used. Considering the hypoglycemic effect of *J. regia* leaves in present study and to obtain better results, we suggest the efficacy of higher doses investigated in future clinical trials.

## Competing interests

The authors declare that they have no competing interests.

## Authors’ contributions

LJ: Designer and project manager, Sample collection, Article writing. HFH: Sample collection, Doing Statistical Analysis. SH: Designer and project manager. KM: Statistical advisor. BL: Cost of testing. AN: Sample collection. KN: Doing Statistical Analysis. All authors read and approved the final manuscript.
